# Application of self-assembly peptides targeting the mitochondria as a novel treatment for sorafenib-resistant hepatocellular carcinoma cells

**DOI:** 10.1038/s41598-020-79536-z

**Published:** 2021-01-13

**Authors:** Tae Ho Hong, M. T. Jeena, Ok-Hee Kim, Kee-Hwan Kim, Ho Joong Choi, Kyung Hee Lee, Ha-Eun Hong, Ja-Hyoung Ryu, Say-June Kim

**Affiliations:** 1 Department of Surgery, Seoul St. Mary’s Hospital, College of Medicine, The Catholic University of Korea, 222, Banpo-daero, Seocho-gu, Seoul, 06591 Republic of Korea; 2 Catholic Central Laboratory of Surgery, Institute of Biomedical Industry, College of Medicine, The Catholic University of Korea, Seoul, Republic of Korea; 3 Department of Chemistry, Ulsan National Institute of Science and Technology (UNIST), Ulsan, Republic of Korea; 4 Department of Surgery, Uijeongbu St. Mary’s Hospital, College of Medicine, The Catholic University of Korea, Seoul, Republic of Korea

**Keywords:** Cancer, Drug discovery, Diseases, Medical research, Molecular medicine

## Abstract

Currently, there is no appropriate treatment option for patients with sorafenib-resistant hepatocellular carcinoma (HCC). Meanwhile, pronounced anticancer activities of newly-developed mitochondria-accumulating self-assembly peptides (Mito-FF) have been demonstrated. This study intended to determine the anticancer effects of Mito-FF against sorafenib-resistant Huh7 (Huh7-R) cells. Compared to sorafenib, Mito-FF led to the generation of relatively higher amounts of mitochondrial reactive oxygen species (ROS) as well as the greater reduction in the expression of antioxidant enzymes (*P* < 0.05). Mito-FF was found to significantly promote cell apoptosis while inhibiting cell proliferation of Huh7-R cells. Mito-FF also reduces the expression of antioxidant enzymes while significantly increasing mitochondrial ROS in Huh7-R cells. The pro-apoptotic effect of Mito-FFs for Huh7-R cells is possibly caused by their up-regulation of mitochondrial ROS, which is caused by the destruction of the mitochondria of HCC cells.

## Introduction

Hepatocellular carcinoma (HCC) has very poor prognosis due to the usual detection at the advanced stages of the disease, the presence of underlying cirrhosis, and paucity of effective treatments. Although various chemotherapeutic agents have been applied for the treatment of advanced HCC, none was able to prolong survival until the mid-2000s. Subsequently, sorafenib has demonstrated significant improvement in survival compared to placebo in phase 3 clinical studies in advanced HCC patients^1^. Sorafenib is a tyrosine kinase inhibitor that was approved by the United States Food and Drug Administration (FDA) in 2007 as the first-line systemic therapy for HCC^2,3^. Sorafenib acts on a variety of sites, including vascular endothelial growth factor receptor 1 (VEGFR1), VEGFR2, VEGFR3, platelet-derived growth factor receptor beta (PDGFR-β), and RAF-family kinases^4^. In the SHARP phase 3 trial incorporating 602 HCC patients, sorafenib improved mean overall survival by approximately 2–3 months compared to the placebo group (10.7 vs. 7.9 months; *P* < 0.001)^1,5^. However, sorafenib frequently causes side effects, including diarrhea, hand-foot syndrome, and fatigue, which results in poor patient compliance. The most common reasons for discontinuation of sorafenib are gastrointestinal events (6%), fatigue (5%), and liver dysfunction (5%)^1^. Moreover, resistance to sorafenib is very prevalent; in the SHARP trial, one-quarter of patients experienced resistance^1,5^. Presently, there is no appropriate treatment option for the patients with sorafenib-resistant HCC^6–9^. These limitations with sorafenib have compelled the need to develop novel or supplementary drugs for patients that are adversely affected by sorafenib.

We previously validated the pronounced anticancer properties of a newly developed mitochondria-accumulating phenylalanine dipeptide with triphenyl phosphonium (Mito-FF)^10,11^. Mito-FF accumulates in mitochondria at concentrations that are 500–1000 times higher concentration than that of other spaces, making self-assembly possible by allowing the concentration of Mito-FF to exceed critical aggregation concentration (CAC)^11,12^. Mito-FF consists of diphenylalanine, TPP, and pyrene, Diphenylalanine peptides are essential building blocks that also make up amyloids in Alzheimer's disease and other neurodegenerative diseases. Assembled diphenylalanine peptides form β-sheets, supported by hydrogen bonding and π–π stacking of aromatic residues^13,14^. TPP is a delocalized lipophilic cation that is crucial in the accumulation of Mito-FF in the mitochondria. Since the mitochondrial inner membrane has a negative potential, the positively charged TPP can facilitate the accumulation of Mito-FF in the mitochondrial matrix to levels that exceed the CAC and drive self-assembly. Final constituent of Mito-FF is pyrene that acts as a florescent probe. This study was undertaken to determine the anticancer effects of Mito-FF against sorafenib-resistant HCC cells. If Mito-FF is demonstrated to have significant anticancer effects on sorafenib-resistant HCC cells, it is expected to provide new possibilities for the treatment of patients with sorafenib-resistant HCC.

## Results

### Generation of sorafenib-resistant HCC cells

**Figure 1:**
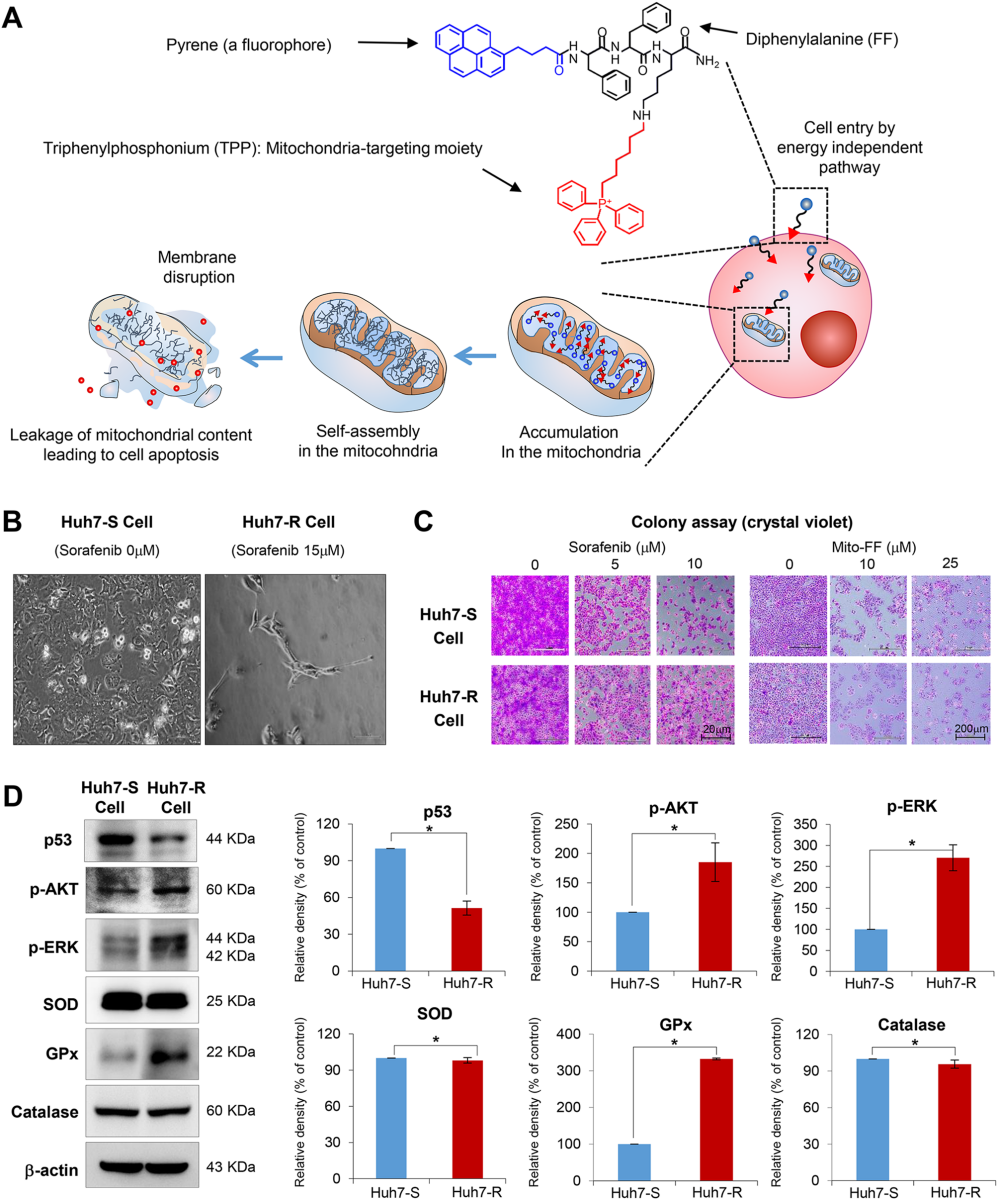
Generation and initial validation of sorafenib-resistant Huh7 cells. (**A**) Structure and mechanism of mitochondria-accumulating phenylalanine dipeptide with triphenyl phosphonium (Mito-FF). Mito-FF consists of diphenylalanine, triphenylphosphonium (TPP), and pyrene (a fluorophore), which serve as β-sheet-forming building blocks, mitochondrial targeting moieties, and fluorescent probes, respectively. Mito-FFs are expected to be accumulated in the mitochondrial matrix after passing the mitochondrial inner membrane, wherein they are self-assembled to form a fibrous structure, eventually leading to apoptotic cell death by destructing mitochondria in tumor cells. (**B**) Microphotographs showing Huh7 cells with no sorafenib [Left] and 15 μM sorafenib treatment [Right], respectively. Huh7 cells that can survive above 15 μM sorafenib were determined as Huh7-R cells. (**C**) Colony assay for the determination of cell survival following the administration of different concentrations of sorafenib and Mito-FF, respectively. (**D**) Western blot analysis [Left] for the comparison of p53, cell proliferation-related proteins (p-AKT and p-ERK) and antioxidant proteins (SOD, GPx, and catalase) in each type of HCC cells. [Right] Relative densities of the markers in each group. Relative densities of individual markers had been quantified using Image J software (http://imagej.nih.gov/ij/download.html ) and then were normalized to that of β-actin in each group. Values are presented as mean ± standard deviation of three independent experiments. *P < 0.05. Abbreviations: GPx, glutathione peroxidase; Huh7-S cell; sorafenib-sensitive Huh7 cell; Huh7-R cell; sorafenib-resistant Huh7 cell; Mito-FF; mitochondria-accumulating phenylalanine dipeptide with triphenyl phosphonium; p-ERK, phosphorylated extracellular signal-regulated kinase; SOD, superoxide dismutase.

Mito-FF is a type of self-assembly peptide that targets mitochondria, and consists of diphenylalanine, TPP, and pyrene (a fluorophore) (Fig. 1A). Mito-FFs are expected to be accumulated in the mitochondrial matrix after passing the mitochondrial inner membrane, wherein they are self-assembled to form a fibrous structure, eventually leading to apoptotic cell death by destructing mitochondria in tumor cells.

To generate sorafenib-resistant strains, Huh7 HCC cells were grown in vitro at increasing doses of sorafenib for a total of 8 months, starting at a sorafenib concentration of 0.5 µmol/L and increasing up to 15 µmol/L. The sorafenib-resistant Huh7 (Huh7-R) cells were able to survive up to the concentration of 15 μM sorafenib (Fig. 1B). Colony assay was performed for the determination of cell survival following the administration of different concentrations of sorafenib and Mito-FF, respectively. When sorafenib (5–10 μM) was administered, sorafenib-sensitive Huh7 (Huh7-S) cells hardly formed colonies above 10 μM sorafenib. However, when Mito-FF (10–25 μM) was administered, no significant difference was found in the colony formation between Huh7-S/R cells (Fig. 1C). Following administration of Mito-FF, cell morphology was changed rapidly at 25 μM Mito-FF in both Huh7-S/R cells, and apoptosis is rapidly induced at 50 μM Mito-FF. We thus think that 50 μM Mito-FF is the optimal concentration for inducing cell apoptosis in both Huh7-S/R cells. Subsequently, western blot analysis was complemented to compare the expression of p53, cell proliferation-related proteins (p-AKT and p-ERK) and antioxidant proteins (SOD, GPx, and catalase) in each type of HCC cells (Fig. 1D). Huh7-R cells showed significantly lower expression of p53, p-AKT and p-ERK than Huh7-S cells (*P* < 0.05). Of antioxidant proteins, GPx was expressed higher in Huh7-R cells than in Huh7-S cells (*P* < 0.05).

### Effects of Mito-FF on the viability of sorafenib-resistant Huh7 cells

**Figure 2:**
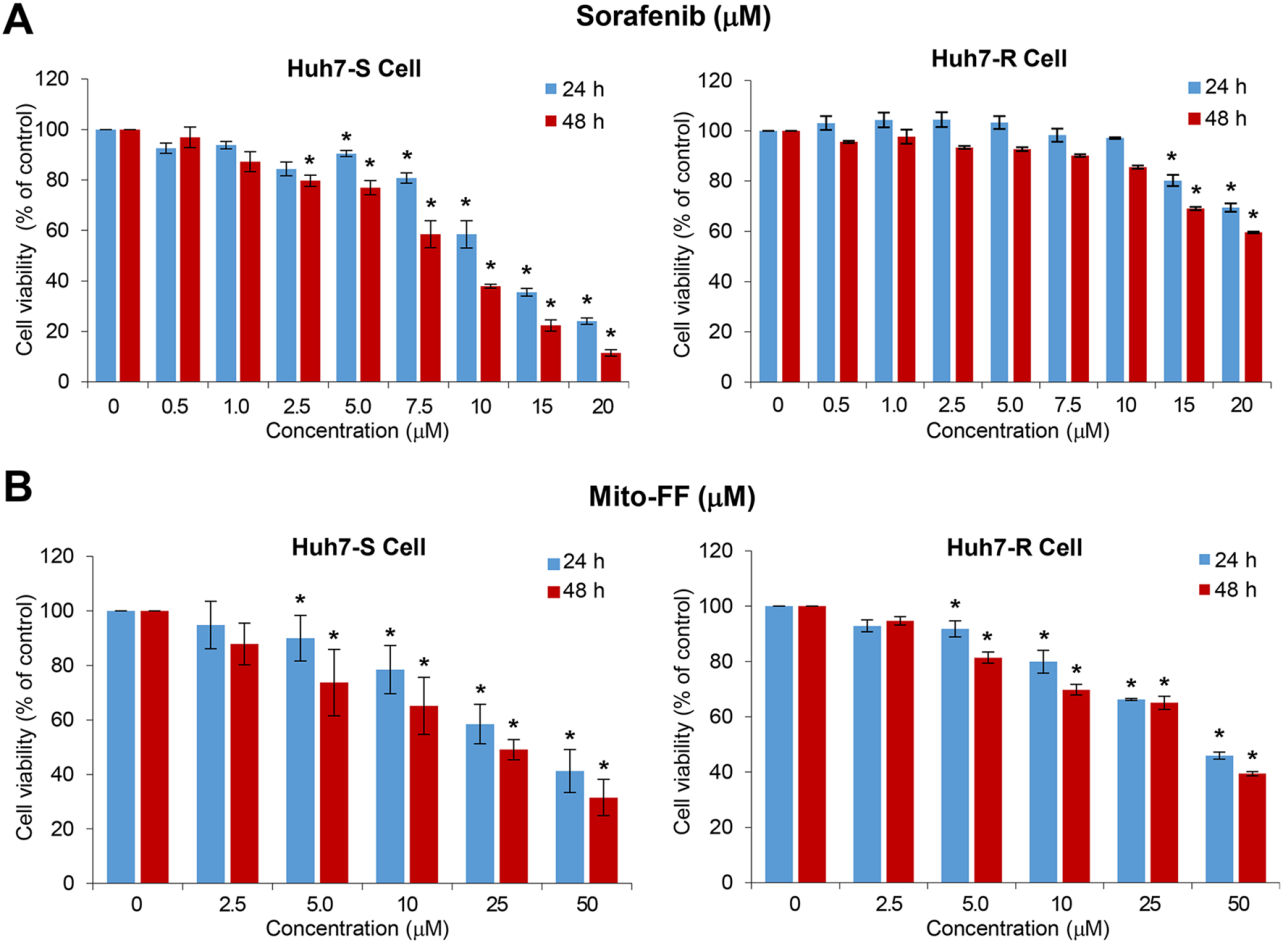
Effects of Mito-FF on the viability of sorafenib-resistant Huh7 cells. (**A**) Cell viability assay of sorafenib-sensitive [Left]/resistant [Right] Huh7 cells following sorafenib treatment. (**B**) Cell viability assay of sorafenib-sensitive [Left]/resistant [Right] Huh7 cells following Mito-FF treatment. Values are presented as mean ± standard deviation of three independent experiments. *P < 0.05. Abbreviation: Huh7-S cell; sorafenib-sensitive Huh7 cell; Huh7-R cell; sorafenib-resistant Huh7 cell; Mito-FF; mitochondria-accumulating phenylalanine dipeptide with triphenyl phosphonium.

Cell viability test was performed for the determination of the effects of sorafenib and Mito-FF on the viability of Huh7-S/R cells, respectively. After sorafenib treatment, viability of Huh7-S cells decreased in concentration- and time-dependent manners (Fig. 2A). However, the viability of Huh7-R cells did not decrease until they were treated with high concentrations (≥ 15 μM). By contrast, the treatment of Mito-FF resulted in the significant reduction of cell viability, irrespective of sorafenib sensitivity, in concentration- and time-dependent manners (*P* < 0.05) (Fig. 2B).

### Effects of Mito-FF on the expression of apoptosis-related and proliferation-related proteins

**Figure 3:**
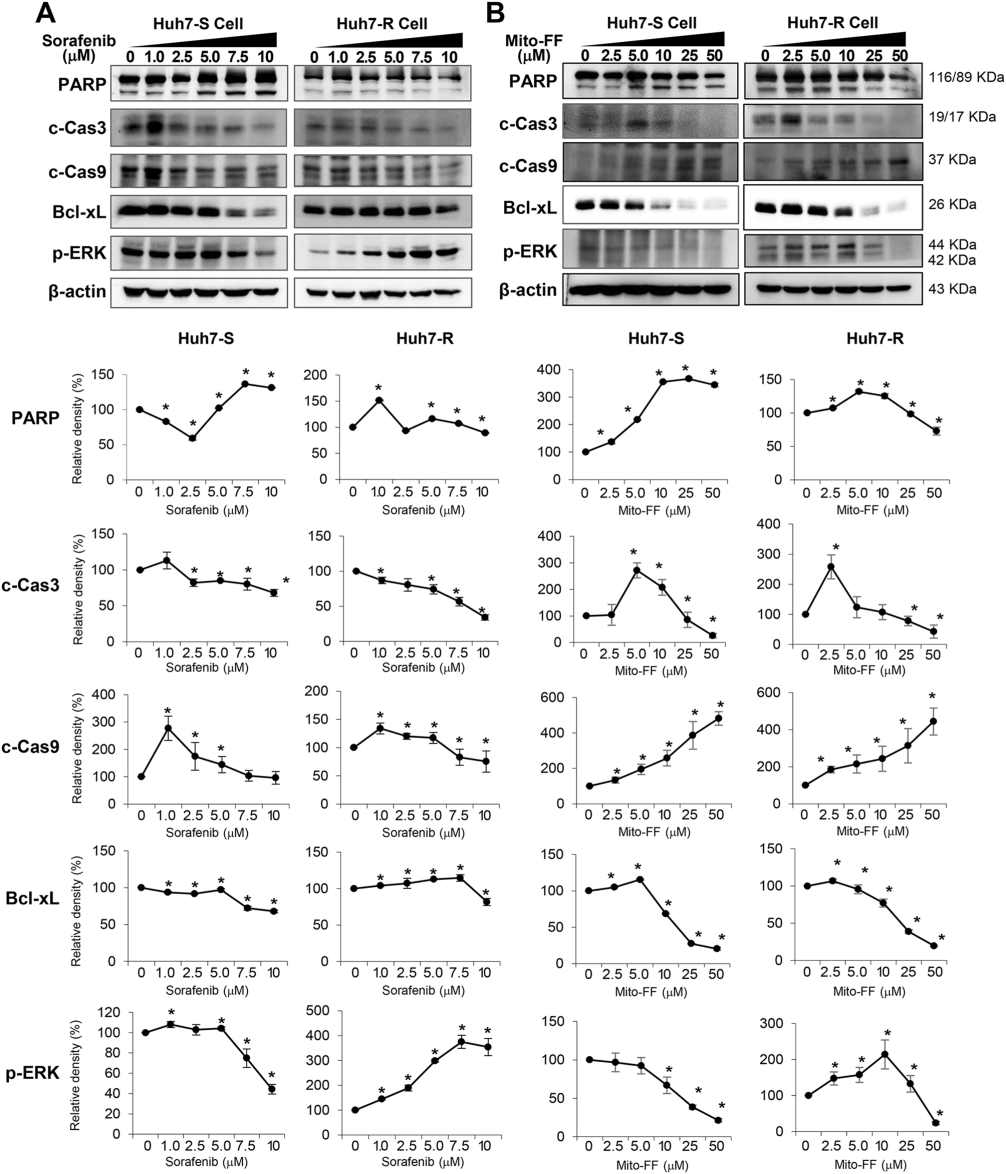
Effects of Mito-FF on the expression of apoptosis-related and proliferation-related proteins. (**A**) (Top) Western blot analysis showing the expression of proteins related with apoptosis and proliferation of sorafenib-sensitive [Left] /resistant [Right] Huh7 cells after sorafenib treatment. (Bottom) Relative densities of the markers in each group. (**B**) (Top) Western blot analysis showing the expression of proteins related with apoptosis and proliferation of sorafenib-sensitive [Left]/resistant [Right] Huh7 cells after Mito-FF treatment. (Bottom) Relative densities of the markers in each group. Relative densities of individual markers had been quantified using Image J software (http://imagej.nih.gov/ij/download.html) and then were normalized to that of β-actin in each group. Values are presented as mean ± standard deviation of three independent experiments. *P < 0.05. Abbreviations: BAX, Bcl-2-like protein 4; Bcl-xL, B-cell leukemia-extra large; c-Cas3, cleaved caspase-3; c-Cas9, cleaved caspase-9; Huh7-S cell; sorafenib-sensitive Huh7 cell; Huh7-R cell; sorafenib-resistant Huh7 cell; Mito-FF; mitochondria-accumulating phenylalanine dipeptide with triphenyl phosphonium; PARP, poly-ADP (adenosine diphosphate)-ribose polymerase; p-ERK, phosphorylated extracellular signal-regulated kinase.

Western blot analysis determined the expression of proteins related with apoptosis (PARP, cleaved caspase-3, cleaved caspase-9, and Bcl-xL) and proliferation (p-ERK) in each type of HCC cells following sorafenib treatment. Overall, the expression of individual markers was similar between Huh7-S and Huh7-R cells after sorafenib treatment except for the expression of PARP and p-ERK. Although sorafenib generally reduced the expression of pro-apoptotic proteins dose-dependently, the expression of PARP in Huh7-S cells was increased since sorafenib was given more than 2.5 μM. On the other hand, in Huh7-R cells, the expression of p-ERK was increased despite increasing sorafenib concentration (Fig. 3A).

Subsequently, the expression of these markers was determined following Mito-FF treatment. Overall, the patterns of expression of these markers did not show significant difference between Huh7-S/R cells. More specifically, the expression of pro-apoptotic markers increased to a certain concentration in a dose-dependent manner, while the expression of anti-apoptotic markers decreased dose-dependently. In addition, the expression of p-ERK was also reduced in a dose -dependent manner. Taken altogether, Mito-FF seems to promote cell apoptosis while inhibiting cell proliferation of Huh7 cells, regardless of whether they are Huh7-S or Huh7-R cells (Fig. 3B).

### Effects of Mito-FF on the apoptotic cell death

**Figure 4:**
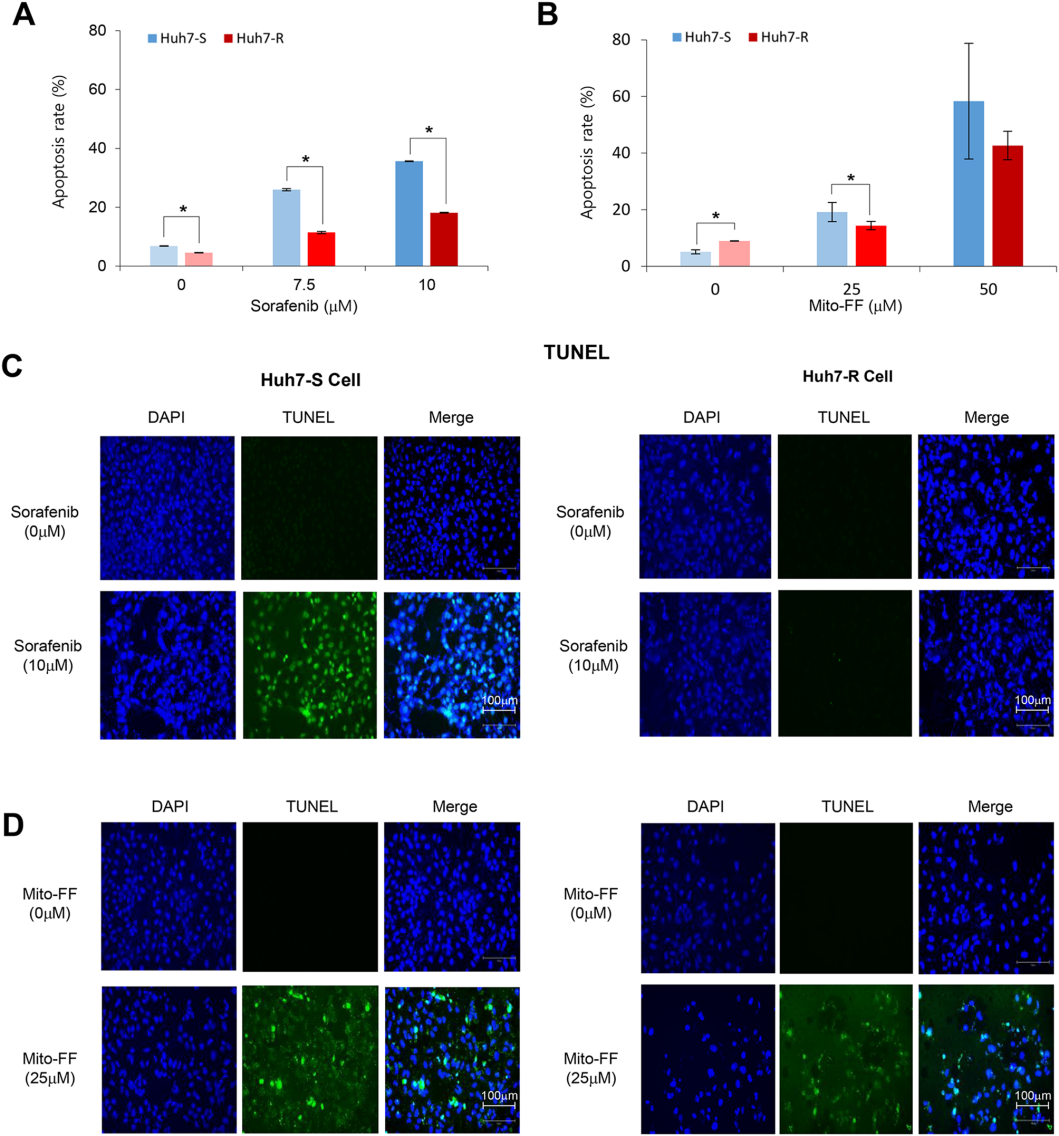
Effects of Mito-FF on the apoptotic cell death. (**A**) Quantification of flow cytometric analysis of sorafenib-sensitive/resistant Huh7 cells after AnnexinV/PI staining following sorafenib treatment. The proportion of apoptosis cells (%) were expressed as the total percentage of Annexin V-positive/PI-negative cells. (**B**) Quantification of flow cytometric analysis of sorafenib-sensitive/resistant Huh7 cells after AnnexinV/PI staining following Mito-FF treatment. (**C**) TUNEL assay to determine apoptosis of sorafenib-sensitive [left] /resistant [right] Huh7 cells by sorafenib, respectively. Compared to Huh7-S cells, fewer TUNEL-positive cells were found in Huh7-R cells after treatment with 10 μM sorafenib. (**D**) TUNEL assay to determine apoptosis of sorafenib-sensitive [left] /resistant [right] Huh7 cells by Mito-FF, respectively. Similar number of TUNEL-positive cells were found in Huh7-R cells as well as Huh7-S cells after treatment with 25 μM Mito-FF. Values are presented as mean ± standard deviation of three independent experiments. *P < 0.05. Abbreviations: Huh7-S cell; sorafenib-sensitive Huh7 cell; Huh7-R cell; sorafenib-resistant Huh7 cell; Mito-FF; mitochondria-accumulating phenylalanine dipeptide with triphenyl phosphonium.

Cell apoptosis was further determined by flow cytometry of AnnexinV/PI stained cells of each type (Supplementary Information). Sorafenib dose-dependently elevated the population of Annexin V-positive cells (early and late apoptotic cells) of both cell types of Huh7 cells. When comparing with Huh7-S/R cells, Huh7-R cells had significantly lower apoptotic cell populations than Huh7-S cells at the same concentration of sorafenib (*P* < 0.05) (Fig. 4A). Similar to sorafenib, Mito-FF dose-dependently elevated the population of Annexin V-positive cells of both Huh7-S/R cells (Fig. 4B). Especially, contrasted by sorafenib treatment, there showed no significant difference in the populations of apoptotic cell fraction according to Huh7-S/R cells following higher concentrations of Mito-FF (50 μM), suggestive of higher induction of apoptotic cell death in Huh7-R cells by Mito-FF.

Subsequently, terminal deoxynucleotidyl transferase-mediated nick end labeling (TUNEL) assay were performed to ascertain the induction of apoptosis in Huh7-S/R cells by sorafenib and Mito-FF, respectively. Compared to Huh7-S cells, fewer TUNEL-positive cells were found in Huh7-R cells after treatment with 10 μM sorafenib (Fig. 4C). By contrast, similar number of TUNEL-positive cells was found in Huh7-R cells as well as Huh7-S cells after treatment with 25 μM Mito-FF (Fig. 4D).

### Effects of Mito-FF on the expression of antioxidant enzymes

**Figure 5:**
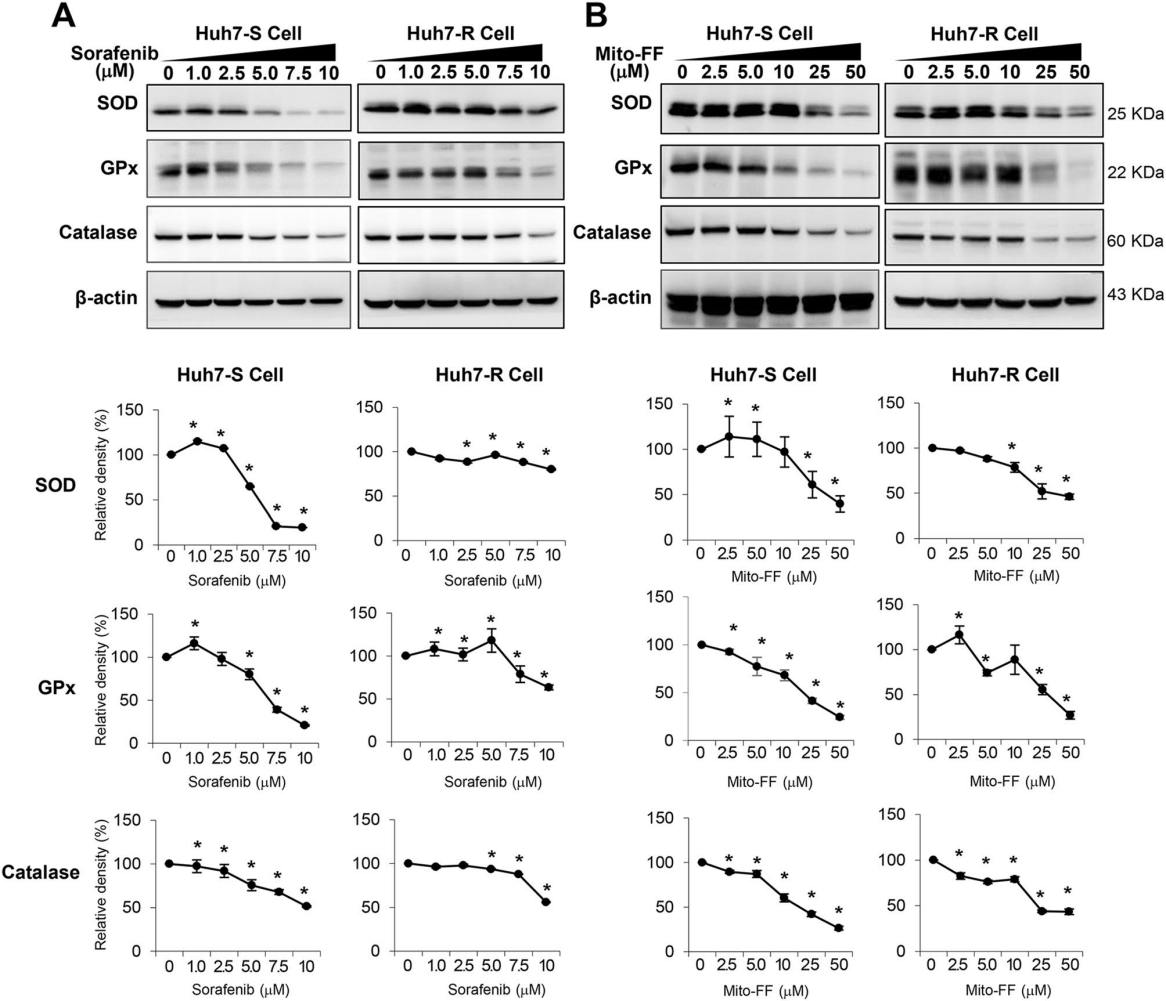
Effects of Mito-FF on the expression of antioxidant proteins. (**A**) (Top) Western blot analysis showing the expression of antioxidant proteins of sorafenib-sensitive [Left] /resistant [Right] Huh7 cells after sorafenib treatment. (Bottom) Relative densities of the markers in each group. (**B**) (Top) Western blot analysis showing the expression of antioxidant proteins of sorafenib-sensitive [Left] /resistant [Right] Huh7 cells after Mito-FF treatment. (Bottom) Relative densities of the markers in each group. Relative densities of individual markers had been quantified using Image J software (http://imagej.nih.gov/ij/download.html) and then were normalized to that of β-actin in each group. Values are presented as mean ± standard deviation of three independent experiments. *P < 0.05. Abbreviations: GPx, glutathione peroxidase; Huh7-S cell; sorafenib-sensitive Huh7 cell; Huh7-R cell; sorafenib-resistant Huh7 cell; Mito-FF; mitochondria-accumulating phenylalanine dipeptide with triphenyl phosphonium; SOD, superoxide dismutase.

Next, the expression of antioxidant proteins was investigated in each type of Huh7 cells following each treatment. Sorafenib reduced the expression of antioxidant proteins (SOD, GPx, and catalase) in a dose-dependent manner. When comparing with Huh7-S/R cells, Huh7-R cells required the higher concentration of sorafenib to reduce the expression of these antioxidant proteins (*P* < 0.05) (Fig. 5A). Likewise, Mito-FF reduced the expression of antioxidant proteins of Huh7-S/R cells in a dose-dependent manner. However, there was no significant difference in the expression of these proteins between Huh7-S/R cells (Fig. 5B).

### Effects of Mito-FF on the mitochondrial ROS levels

**Figure 6:**
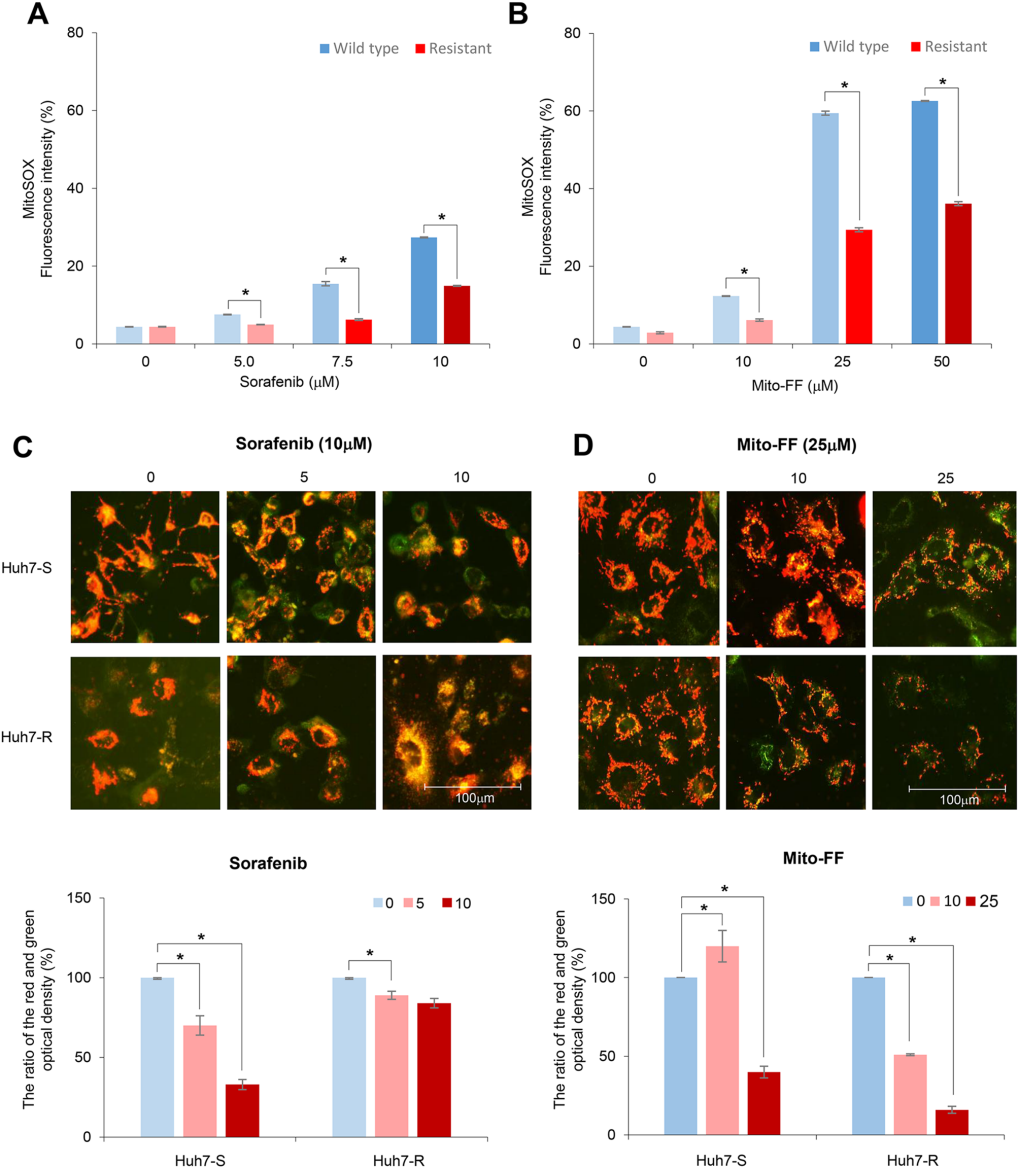
Anticancer effects of Mito-FF in relation with oxidative stress. (**A**) Quantification of mitochondrial ROS levels of Huh7 cells according to the increasing concentration of sorafenib by flow cytometric analysis. Mitochondrial ROS was determined by quantification of the red fluorescence that emits when MitoSOX Red reagent (a mitochondrial superoxide indicator) is oxidized by superoxide in the mitochondria. (**B**) Quantification of mitochondrial ROS levels of Huh7 cells according to the increasing concentration of Mito-FF by flow cytometric analysis. (**C**) [Top] Determination of mitochondrial membrane potential (MMP) using JC-1 dye in Huh7 S/R cells following sorafenib treatment. In normal cells, JC-1 fluorescent dyes can accumulate in the matrix of mitochondria, producing red fluorescence. However, when the MMP declined, JC-1 cannot gather in the matrix so that JC-1 exist in the matrix as monomer, producing green fluorescence. [Bottom] The ratio of the red (OD1) and green (OD2) optical density. As the concentration of sorafenib increased, the ratio in the Huh7-S cells declined; the ratio in the Huh7-R cells was unchanged. (**D**) [Top] Determination of MMP using JC-1 dye in Huh7 S/R cells following Mito-FF treatment. [Bottom] The ratio of the red and green optical density. As the concentration of Mito-FF increased, the ratios declined in both Huh7-S/R cells. Values are presented as mean ± standard deviation of three independent experiments. *P < 0.05. Abbreviation: Huh7-S cell; sorafenib-sensitive Huh7 cell; Huh7-R cell; sorafenib-resistant Huh7 cell; Mito-FF; mitochondria-accumulating phenylalanine dipeptide with triphenyl phosphonium.

MitoSOX Red reagent (a mitochondrial reactive oxygen species [ROS] indicator) is oxidized in the mitochondria by superoxide, resulting in producing red fluorescence^15^. Thus, mitochondrial ROS levels in the targeted cells can be determined by flow cytometric analysis of the MitoSOX stained cells (Supplementary Information). MitoSOX-based flow cytometry demonstrated that sorafenib increased the mitochondrial ROS levels of each type of HCCs in a dose-dependent manner. When comparing with Huh7-S/R cells, Huh7-R cells displayed less mitochondrial ROS than Huh7-S cells (*P* < 0.05), especially at higher concentrations of sorafenib (≥ 5 μM) (Fig. 6A). Likewise, Mito-FF increased the mitochondrial ROS levels of each type of HCCs in a dose-dependent manner. When comparing Huh7-S/R cells, Huh7-R cells displayed lower levels of mitochondrial ROS than Huh7-S cells (*P* < 0.05), especially at higher concentrations of Mito-FF (≥ 25 μM) (Fig. 6B).

Subsequently, we performed JC-1 assay to determine the alternations of mitochondrial membrane potential (MMP) following each treatment. In the normal cells, JC-1 fluorescent dyes accumulate in the matrix of mitochondria, producing red fluorescence. However, when MMP is reduced—for instant, as a result of the induction of apoptosis, JC-1 cannot gather the matrix so that JC-1 exists in the matrix as a monomer, producing green fluorescence. Sorafenib treatment to Huh7-S cells led to some of JC-1 fluorescence color change from red to green along with the increasing concentration of sorafenib, suggesting that MMP declined along with the increasing concentration of sorafenib; sorafenib treatment to Huh7-R cells did not (Fig. 6C). In addition, Mito-FF treatment to both Huh7-S/R cells led to the color change from red to green—to some extent—with the increasing concentration of Mitro-FF, suggesting that MMP declined along with the increasing concentration of Mito-FF (Fig. 6D).

### Determination of the action mechanism of Mito-FF in relation with ROS

**Figure 7:**
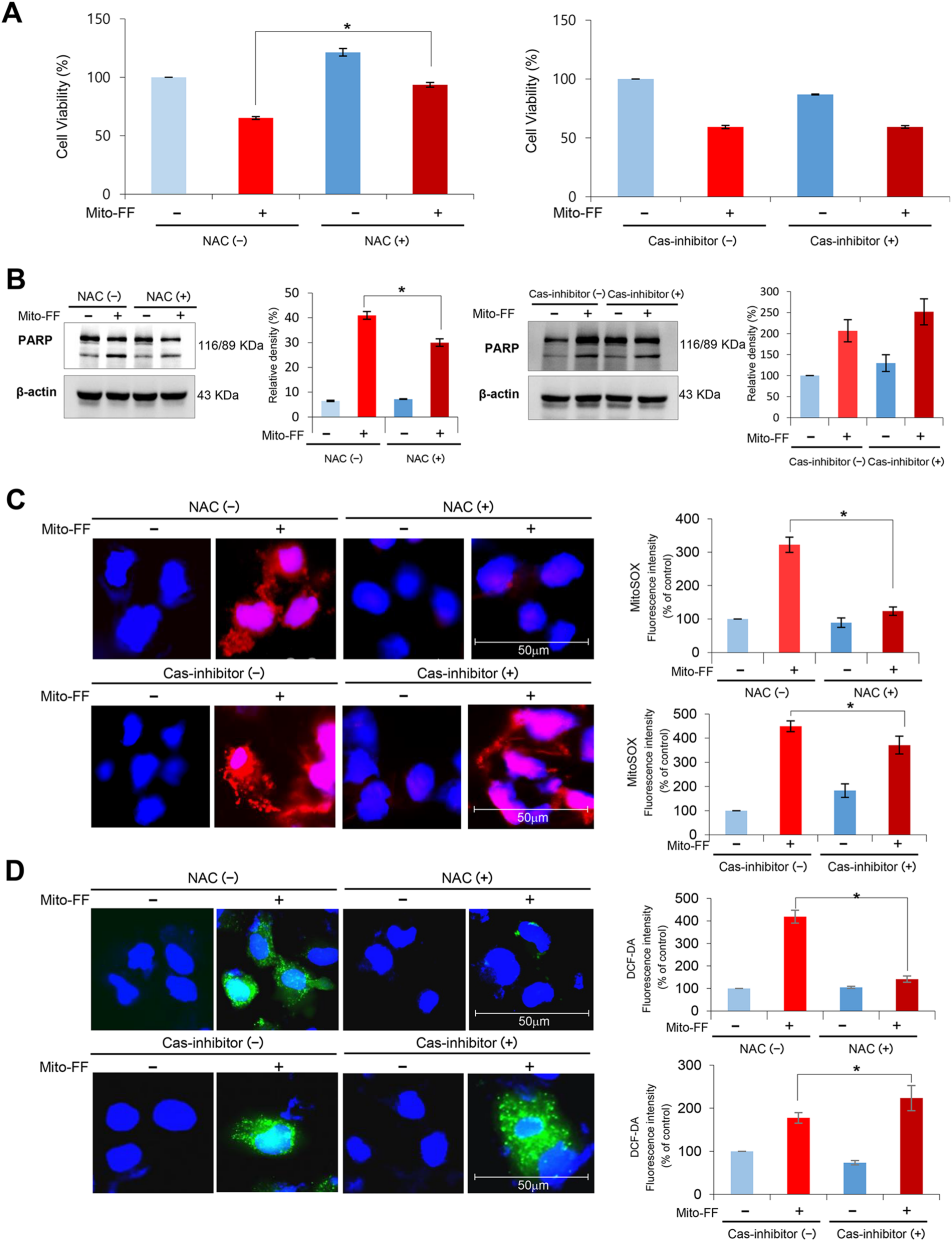
Determination of the action mechanism of Mito-FF in relation with ROS and caspase signaling. (**A**) Cell viability assay of sorafenib-resistant Huh7 cells that were treated with Mito-FF as well as *N*-acetyl-l-cysteine (NAC; ROS inhibitor) [Left] or Z-VAD-fmk (a caspase inhibitor) [Right]. Addition of NAC with Mito-FF-treated Huh7-R cells significantly increased the cell viability; addition of Z-VAD-fmk did not. (**B**) Western blot analysis showing the expression of PARP (a pro-apoptotic marker) in Huh7-R cells treated either with NAC or Z-VAD-fmk. The addition of NAC significantly reduced the expression of PARP in the Mito-FF treated Huh7-R cells; the addition of Z-VAD-fmk did not. (**C**) MitoSOX immunofluorescence of sorafenib-resistant Huh7 cells according to the addition of NAC and Z-VAD-fmk, respectively. Percentages of immunofluorescent areas were measured using NIH image J (http://imagej.nih.gov/ij/download.html) and expressed as relative values to those in control cells. (**D**) DCF-DA immunofluorescence of sorafenib-resistant Huh7 cells according to the addition of NAC and Z-VAD-fmk, respectively. Percentages of immunofluorescent areas were measured using NIH image J (http://imagej.nih.gov/ij/download.html) and expressed as relative values to those in control cells. Values are presented as mean ± standard deviation of three independent experiments. *P < 0.05. Abbreviation: DCF-DA, 2′,7′–dichlorofluorescin diacetate, Huh7-S cell; sorafenib-sensitive Huh7 cell; Huh7-R cell; sorafenib-resistant Huh7 cell; Mito-FF; mitochondria-accumulating phenylalanine dipeptide with triphenyl phosphonium; NAC, *N*-acetyl-l-cysteine; PARP, poly-ADP (adenosine diphosphate)-ribose polymerase; ROS, reactive oxygen species.

The mechanism of action of Mito-FF on the Huh7-R cells was investigated in relation with mitochondrial ROS. Firstly, cell viability assay of Mito-FF treated Huh7-R cells after treating either with or *N*-acetyl-l-cysteine (NAC; an ROS inhibitor) or Z-VAD-fmk (a caspase inhibitor) was performed. Addition of NAC with Mito-FF-treated Huh7-R cells significantly increased the cell viability (*P* < 0.05); addition of Z-VAD-fmk did not. These results suggest that Mito-FF decreases cell viability of HCC cells by increasing ROS caspase-independently (Fig. 7A). Next, the expression of PARP (an apoptotic marker) in Huh7-R cells treated either with NAC or Z-VAD-fmk was determined by Western blot analysis. The addition of NAC significantly reduced the expression of PARP in the Mito-FF treated Huh7-R cells (*P* < 0.05); the addition of Z-VAD-fmk did not (Fig. 7B). These results suggest that Mito-FF also promotes the apoptosis of Huh7-R cells by increasing mitochondrial ROS. Subsequent MitoSOX (Fig. 7C) and DCF-DA (Fig. 7D) immunofluorescences support the pivotal role of mitochondrial and intracellular ROS in the mechanism of Mito-FF, respectively. Specifically, it was found that addition of NAC to Mito-FF-treated Huh7-R cells significantly reduced the levels of mitochondrial and intracellular ROS, respectively (*P* < 0.05); addition of Z-VAD-fmk did not.

**Figure 8:**
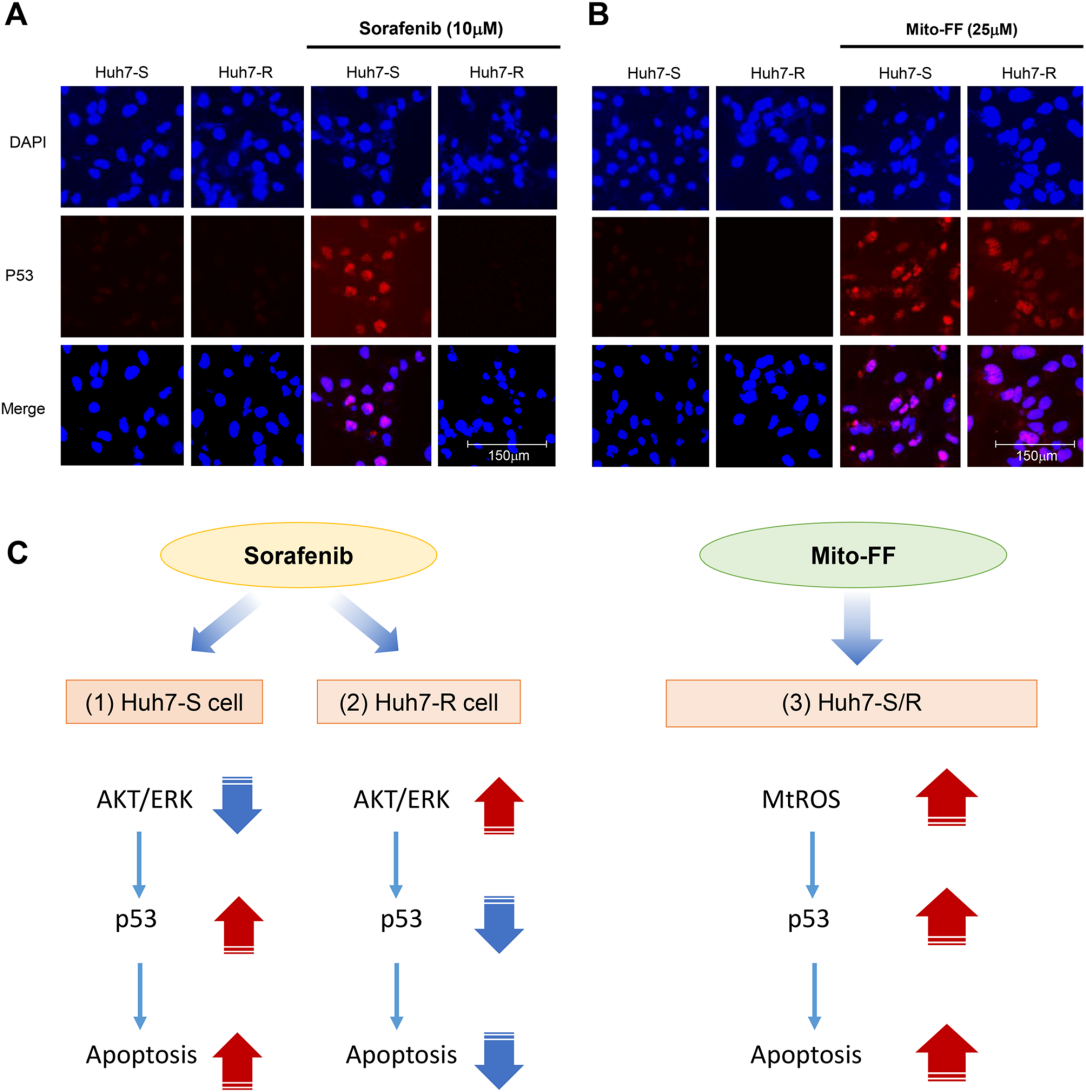
Determination of action mechanism of Mito-FF in relation with ROS and p53. (**A**) Immunofluorescent staining of p53 in Huh-S/R cells before and after sorafenib treatment. Following sorafenib treatment to Huh7-S cells, there was an increase in the percentage of cells with positive nuclear staining for p53; following sorafenib to Huh7-S cells, there was not. (**B**) Immunofluorescent staining of p53 in Huh-S/R cells before and after Mito-FF treatment. There was an increase in the percentage of cells with positive nuclear staining for p53following Mito-FF treatment to both Huh7-S/R cells. (**C**) Possible mechanism of sorafenib and Mito-FF in relation with mitochondrial ROS and p53. Sorafenib treatment decreases AKT/ERK in Huh7-S cells and thus increases the expression of p53, cumulating in increasing apoptosis. Sorafenib treatment did not increase apoptosis in Huh7-R cells—because AKT/ERK was increased in Huh7-R cells and thus p53 was decreased. Treating Mito-FF to Huh7-S/R cells, leads to a significant increase of mitochondrial ROS, by way of evoking mitochondrial dysfunction due to the accumulation and self-assembly in the mitochondrial matrix, resulting in increased expression of p53, which prompts apoptosis.

Next, in order to investigate the role of p53 in the mechanism of Mito-FF, the expression of p53 according to each treatment was determined by immunofluorescence. Addition of sorafenib to Huh7-S cells significantly increased the percentage of cells with positive nuclear staining for p53; addition of sorafenib to Huh7-R cells did not (Fig. 8A). However, following Mito-FF treatment, there was an increase in the percentage of cells with positive nuclear staining for p53—regardless of Huh7-S or Huh7-R cells (Fig. 8B).

Taken altogether, the mechanism of Mito-FF can be postulated as follows (Fig. 8C). Sorafenib treatment could decrease AKT/ERK in Huh7-S cells and thus increases the expression of p53, favoring apoptosis. By contrast, sorafenib treatment could not increase apoptosis in Huh7-R cells—because they has the higher expression of AKT/ERK that inactivates p53. On the other hand, treating Mito-FF to Huh7-S/R cells, could substantially increase mitochondrial ROS, by evoking mitochondrial dysfunction due to the accumulation and self-assembly in the mitochondrial matrix, and thereby could increase the expression of p53, ultimately resulting in enhanced apoptosis, regardless of Huh7-S or Huh7-R cells.

## Discussion

Sorafenib is the only globally available, molecularly targeted drug for the treatment of advanced HCC^16,17^. However, its use is hindered by very limited effectiveness, higher side effects, and a broader population of resistance. In this study, we investigated the therapeutic effect of the novel anticancer drug—Mito-FF—in sorafenib-resistant HCC cells. Mito-FF is one of self-assembly peptide targeting mitochondria. Previous investigations validated that Mito-FFs accumulate in mitochondrial matrix, wherein they are self-assembled, evoking mitochondrial dysfunction^10,11,18^. This is the first validation of anticancer effect of Mito-FF in the HCC cells. Regardless of sorafenib resistance, treatment with Mito-FF significantly reduced cell viability of Huh7 cells; significantly inhibited the proliferation of Huh7 cells; and significantly increased the proportion of apoptotic cells of Huh7 cells.

In recent decades, nanoscience has progressed to become a relevant therapeutic approach. In 2005, the FDA approved the abraxane as a nanomedicine for metastatic breast cancer, advanced non-small cell lung cancer, and metastatic pancreatic cancer^19^. The therapeutic applications of nanostructural assembles of amphiphilic peptides have also been explored as nanomedicines^19–24^. Molecular self-assembly is the process by which materials—especially, peptides—combine to form ordered structures under specific kinetic and thermodynamic conditions^25–27^. This can occur physiologically and pathologically. Physiologic examples of molecular self-assembly include the assembly of phospholipid biological membranes, the association of protein microtubules and microfilaments to provide cellular support, and the formation of the DNA double helix through hydrogen bonding^14,28^. In addition, the representative pathological self-assembly process is the formation of amyloid fibrils in patients with Alzheimer’s disease. For self-assembly to happen, the concentration of the self-assembly molecules must exceed the CAC^11^. It could be very difficult to create an environment in which self-assembly peptides exceed CAC in the total volume of living cells. However, the concentration of self-assembly peptides could exceed CAC in the environment in which they become trapped and are thus unable to be spread outside the organelles.

Mito-FF is more likely to accumulate in cancer cells than in normal cells because cancer cells more frequently have dysfunctional mitochondria and thus have a high negative membrane potential^12,29^. Dysfunctional mitochondria in cancer cells contribute to the loss of mitochondrial proton gradients to a certain extent, which increases the negative potential of mitochondria^11^. Therefore, Mito-FF could accumulate in in cancer cells than in normal cells, because it has triphenylphosphonium (TPP), the delocalized lipophilic cation that targets better to mitochondria of tumor cells with negative membrane potential. Mito-FFs self-assemble in the mitochondrial matrix of cancer cells to form fibrous structures, which aggravates mitochondrial dysfunction. As you shown in Fig. 6, Mito-FF dramatically increased mitochondrial ROS production above a certain concentration (25–50 μM). Excess production of ROS contributes to mitochondrial damage, including mitochondrial DNA damage, membrane disruption, and mitochondrial protein damage, all of which ultimately leads to apoptotic cell death. Mitochondria have several fundamental functions, including producing the energy required by the cell and regulating intracellular oxidative stress by lowering ROS^12,30,31^. In this process, mitochondria maintain cellular homeostasis by regulating ROS production^32,33^. Thus, destruction of mitochondria inevitably leads to an increase in ROS and a decrease in antioxidant enzymes.

The pro-apoptotic effect of Mito-FF appears to be more pronounced in the Huh7-S cells than in the Huh7-R cells. Increased oxidative stress by Mito-FF was more pronounced in the Huh7-S cells than in the Huh7-R cells. This difference is thought to be due to the altered characteristics of Huh7-R cells. One of the key metabolic alterations in the sorafenib resistant cells is to increase antioxidant capacity^34,35^. You et al.^36^ performed a global untargeted metabolomics using sorafenib-resistant cell lines to identify the metabolic alterations relevant to the therapeutic resistance. It was found that both cystathionine-β-synthase and cystathionine-gamma-ligase were substantially increased in the sorafenib-resistant cells. In addition, the majority of enzymes involved in the glutathione synthesis and regeneration were highly expressed in sorafenib-resistant cells Moreover, GPX1, the enzyme responsible for scavenging hydrogen peroxide was upregulated in the resistant cells. The sorafenib-resistant cells exhibited the markedly increased expression of Nrf2, the master transcription factor that regulates the expression of various antioxidant enzymes^37^.

In this study, the mechanism of action of the anti-tumor effect of Mito-FF was investigated. AKT is involved in the regulation of p53 activation: AKT activation leads to the marked suppression of p53^38–40^. p53 is critical for tumor suppression not only during the acute response to cellular stress, such as DNA damage that is characterized by extensive apoptosis, but also during the quiescent status wherein p53 acts like killing or silencing cancer-initiating cells^41^. Sorafenib is a protein kinase inhibitor with activity against multiple protein kinases, including VEGFR, PDGFR, AKT/ERK and RAF kinases. The inhibition of AKT/ERK activity by sorafenib results in activation of p53, which contributes to induction of apoptosis of tumor cells. Sorafenib resistant HCC cells exhibit the characteristics of activated the phosphatidylinositol 3-kinase (PI3K)/AKT signaling pathway^42^, which ultimately limits the induction of apoptosis by the suppression of p53. Our results show that Mito-FF extensively increases mitochondrial and intracellular ROS levels. Excess cellular levels of ROS cause damage to proteins, nucleic acids, lipids, membranes and organelles, which can directly lead to induction of apoptotic process^43,44^. In addition, elevated ROS levels suppress the EGFR/PI3K/AKT signaling pathway^45^, which in turn activates p53^40^, ultimately resulting in the acceleration of apoptotic process.

In conclusion, Mito-FF promoted cell apoptosis while inhibiting cell proliferation of Huh7 cells, regardless of their sorafenib resistance. To reduce the expression of antioxidant enzymes, Huh7-R cells required more sorafenib than Huh7-S cells. However, there was no significant difference in the amount of Mito-FF between Huh7-S and Huh7-R cells to reduce the expression of antioxidant enzymes. Mito-FF increased mitochondrial ROS of Huh7 cells in a concentration-dependent manner even higher than did sorafenib. The results derived from ROS inhibition suggest that Mito-FF promotes the apoptosis of Huh7-R cells by increasing ROS, which is possibly caused by the destruction of the mitochondria of HCC cells. Further investigations are needed to precisely determine whether Mito-FF could be an acceptable treatment for patients with sorafenib-resistant HCC.

## Material and methods

### Chemicals

Sorafenib was purchased from Cayman Chemical (Ann Arbor, MI). NAC and Z-VAD-fmk were purchased from Sigma-Aldrich (St. Louis, MO). MitoSOX was obtained from Thermo Fisher Scientific (Waltham, MA) and 2′,7′-dichlorodihydrofluorescein diacetate (DCF-DA) was purchased from Thermo Fisher Scientific (San Jose, CA).

### Cell culture

Huh7 HCC cells were obtained from Korea Cell Line Bank (KCLB, Seoul, Republic of Korea). Huh7 cells were maintained in DMEM (Thermo Fisher Scientific, Carlsbad, CA). The medium was supplemented with 10% fetal bovine serum (GibcoBRL; Carlsbad, CA) and 1% penicillin–streptomycin (Thermo Fisher Scientific) at 37 °C in a humidified atmosphere with 5% CO_2_ in an incubator. Passage 35 Huh7-S cells were used for the experiment.

### Establishment of sorafenib-resistant HCC cells

To generate sorafenib-resistant cultures, HCC Huh7 cells were grown in vitro in the presence of increasing doses of sorafenib (0.5 µM per week) for a total of 8 months, starting at a sorafenib concentration of 0.5 µmol/L and increasing up to 15 µmol/L. After sorafenib-resistant HCCs were obtained, they were continuously maintained in DMEM contained with 1 µM sorafenib. Passage 51 Huh7-R cells were used for the experiment.

### Cell proliferation assay

Proliferation of sorafenib-sensitive/resistant Huh7 cells were evaluated using EZ-Cytox Cell Proliferation Assay kit (Itsbio, Seoul, Republic of Korea) according to the manufacturer’s instructions.

### Western blot analysis

Sorafenib-sensitive/resistant Huh7 cells were lysed using the EzRIPA Lysis kit (ATTO Corporation, Tokyo, Japan), and quantified using the Bradford reagent (Bio-Rad, Hercules, CA). Proteins were visualized by western blot analysis using the following primary antibodies (1:1000 dilution) and then horseradish peroxidase (HRP)-conjugated secondary antibodies (1:2000 dilution) from Vector Laboratories (Burlingame, CA). Specific immune complexes were detected using the Western Blotting Plus Chemiluminescence Reagent (Millipore, Bedford, MA). We obtained p53 antibody from Abcam (Cambridge, UK) and other antibodies from Cell Signaling Technology (Beverly, MA), including primary antibodies against poly-ADP (adenosine diphosphate)-ribose polymerase (PARP), cleaved caspase 3, cleaved caspase 9, B-cell lymphoma-extra-large (Bcl-xL), BCL2 associated X protein (Bax), phosphorylated extracellular signal-regulated kinase-1 (p-ERK), superoxide dismutase (SOD), glutathione peroxidase (GPx), catalase, β-actin, and HRP-conjugated secondary antibodies.

### MitoSOX staining and JC-1 mitochondrial membrane potential assay

Sorafenib-sensitive/resistant Huh7 cells were cultured on Lab-Tek chamber slides (Thermo Fisher Scientific). After treatment with sorafenib and Mito-FF. respectively, for 24 h, the Huh7 cells were stained with 10 μM MitoSOX or 10 μM DCF-DA reagent at 37 °C for 10 min and were stained with 5 μM JC-1 for 15 min at 37 °C in the dark. Mitochondrial and MMP was observed using the M5000 fluorescence imaging system (EVOS, Invitrogen, CA).

### Immunofluorescence

Huh7-S/R cells were cultured on Lab-Tek chamber slides (Thermo Fisher Scientific). The cells were washed three times with phosphate-buffered saline (PBS), fixed with 4% paraformaldehyde for 30 min, and permeabilized with 0.3% Triton X-100 for 20 min. After blocking with 1% bovine serum albumin for 1 h at 25 °C, the slides were incubated with the antibodies against p53 (1:100 dilution, Santa cruz) at 4 °C overnight. The slides were washed three times with PBS and incubated with Alexa Fluor 594-conjugated secondary antibodies (1:500 dilution) for 1 h at 25 °C; the nuclei were counter-stained with DAPI-containing VECTASHIELD Mounting Medium (Vector Labs, Burlingame, CA) for 1 min. The samples were observed using a fluorescence imaging system (EVOS U5000; Invitrogen, CA).

### TUNEL assay

TUNEL analysis was performed for the detection of apoptosis in Huh7-S/R cells using the in situ Apoptosis Detection Kit (Takara. Bio, Inc., Japan) following the manufacturer’s instructions. In brief, sample slides were incubated with 50 μl of TUNEL reaction mixture and TdT labeling reaction mix for 1 h at 37 °C in the dark. After being rinsed with PBS three times, the samples were observed using a fluorescence imaging system (EVOS U5000; Invitrogen, CA).

### Quantification of Cell ROS and apoptosis by flow cytometry

To detect ROS and apoptosis, cells were stained with either DCF-DA or Annexin V/PI using the FITC Annexin V apoptosis detection kit (BD Biosciences), respectively. After incubation for 15 min in the dark at 25 °C, the cells were analyzed using a BD FACSCANTO II cytometer (BD Biosciences).

### Quantification of ROS and apoptosis by flow cytometry

To determine intracellular ROS levels and the proportion of cells undergoing apoptotic cell death, Huh7 cells were stained with MitoSOX or Annexin V/propidium iodide (PI), respectively. After incubation for 10 min in the dark at 25 °C, the cells were analyzed using an Attune NxT Acoustic focusing cytometer (Thermo Fisher Scientific).

### Statistical analysis

All data were analyzed with SPSS 11.0 software (SPSS Inc., Chicago, IL) and are presented as mean ± standard deviation. Statistical comparison among groups was determined using Kruskal–Wallis test. Probability values of *P* < 0.05 were regarded as statistically significant.

## Supplementary information

Supplementary Information 1.

## Data Availability

The datasets generated during and/or analysed during the current study are available from the corresponding author on reasonable request.

## Abbreviations

GPx: Glutathione peroxidase
Huh7-S cell: Sorafenib-sensitive Huh7 cell
Huh7-R cell: Sorafenib-resistant Huh7 cell
Mito-FF: Mitochondria-accumulating phenylalanine dipeptide with triphenyl phosphonium
p-ERK: Phosphorylated extracellular signal-regulated kinase
SOD: Superoxide dismutase
BAX: Bcl-2-like protein 4
Bcl-xL: B-cell leukemia-extra large
c-Cas3: Cleaved caspase-3
c-Cas9: Cleaved caspase-9
PARP: Poly-ADP (adenosine diphosphate)-ribose polymerase
NAC: *N*-Acetyl-l-cysteine
ROS: Reactive oxygen species
