# Supplementary material for: Application of self-assembly peptides targeting the mitochondria as a novel treatment for sorafenib-resistant hepatocellular carcinoma cells
Source: Sci Rep. 2021 Jan 13;11:874. doi: 10.1038/s41598-020-79536-z (PMC7806888; doi:10.1038/s41598-020-79536-z)

**Application of self-assembly peptides targeting the mitochondria as a novel treatment for sorafenib-resistant hepatocellular carcinoma cells**

(Running head: Self-assembly peptides targeting sorafenib-resistant HCC cells)

Tae Ho Hong^1,2^, M.T. Jeena^3^, Ok-Hee Kim^1,2^, Kee-Hwan Kim^2,4^, Ho Joong Choi^1,2^, Kyung Hee Lee^1,2^, Ha-Eun Hong^1,2^, Ja-Hyoung Ryu^3^, Say-June Kim^1,2^

^1^Department of Surgery, Seoul St. Mary’s Hospital, College of Medicine, the Catholic University of Korea, Seoul, Republic of Korea

^2^Catholic Central Laboratory of Surgery, Institute of Biomedical Industry, College of Medicine, the Catholic University of Korea, Seoul, Republic of Korea

^3^Department of Chemistry, Ulsan National Institute of Science and Technology (UNIST), Ulsan, Republic of Korea

^4^Department of Surgery, Uijeongbu St. Mary's Hospital, College of Medicine, the Catholic University of Korea, Seoul, Republic of Korea


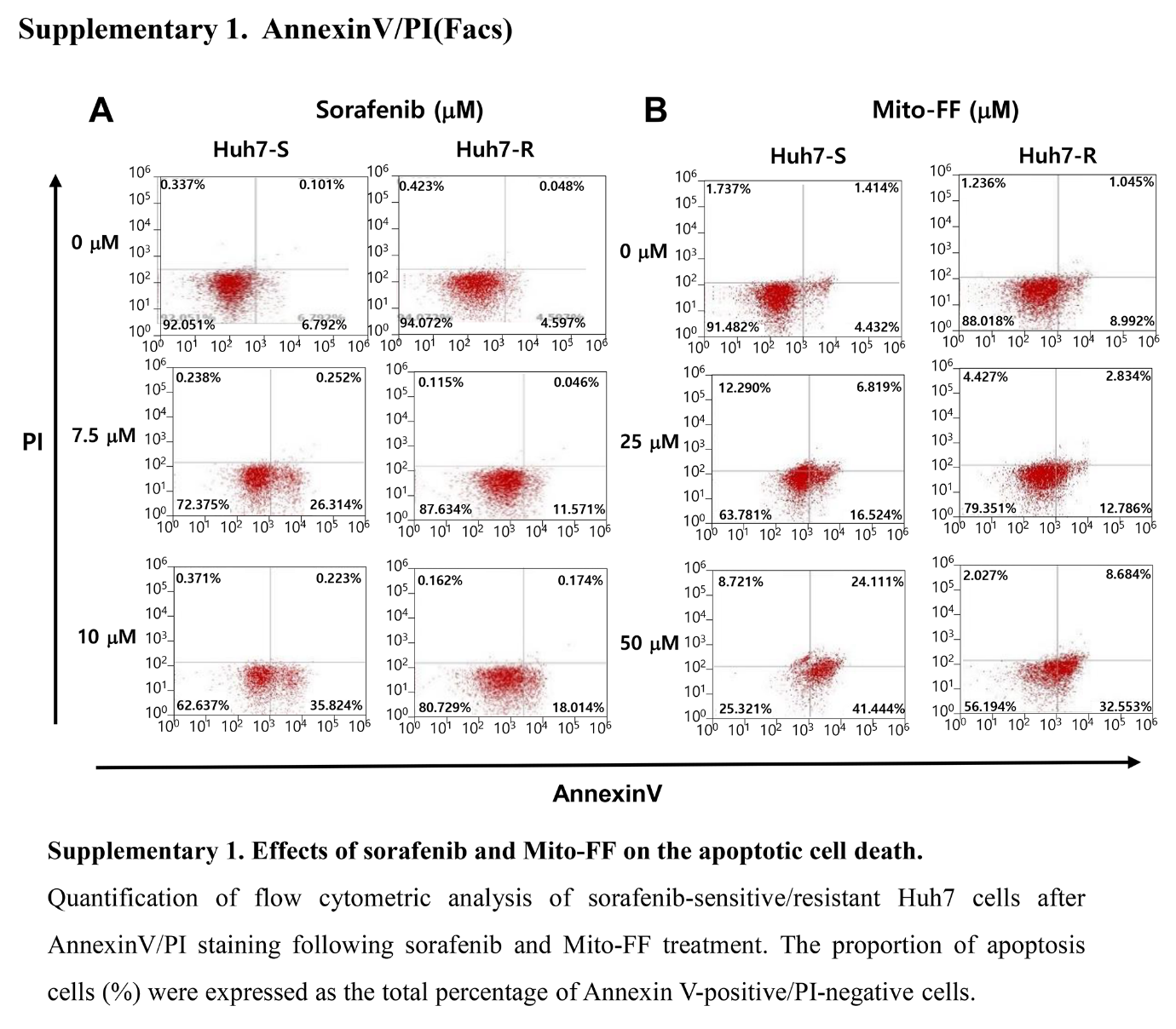


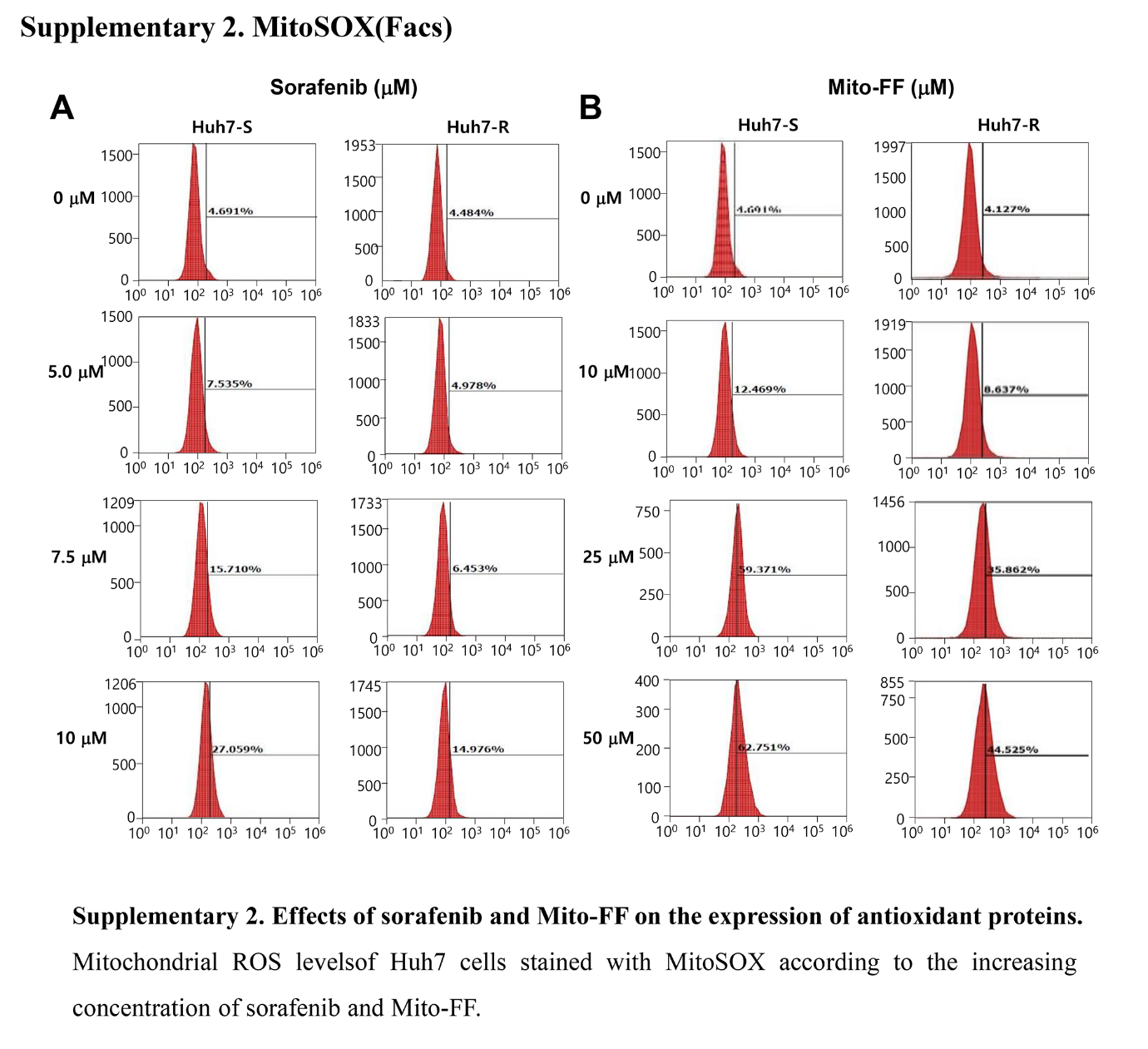


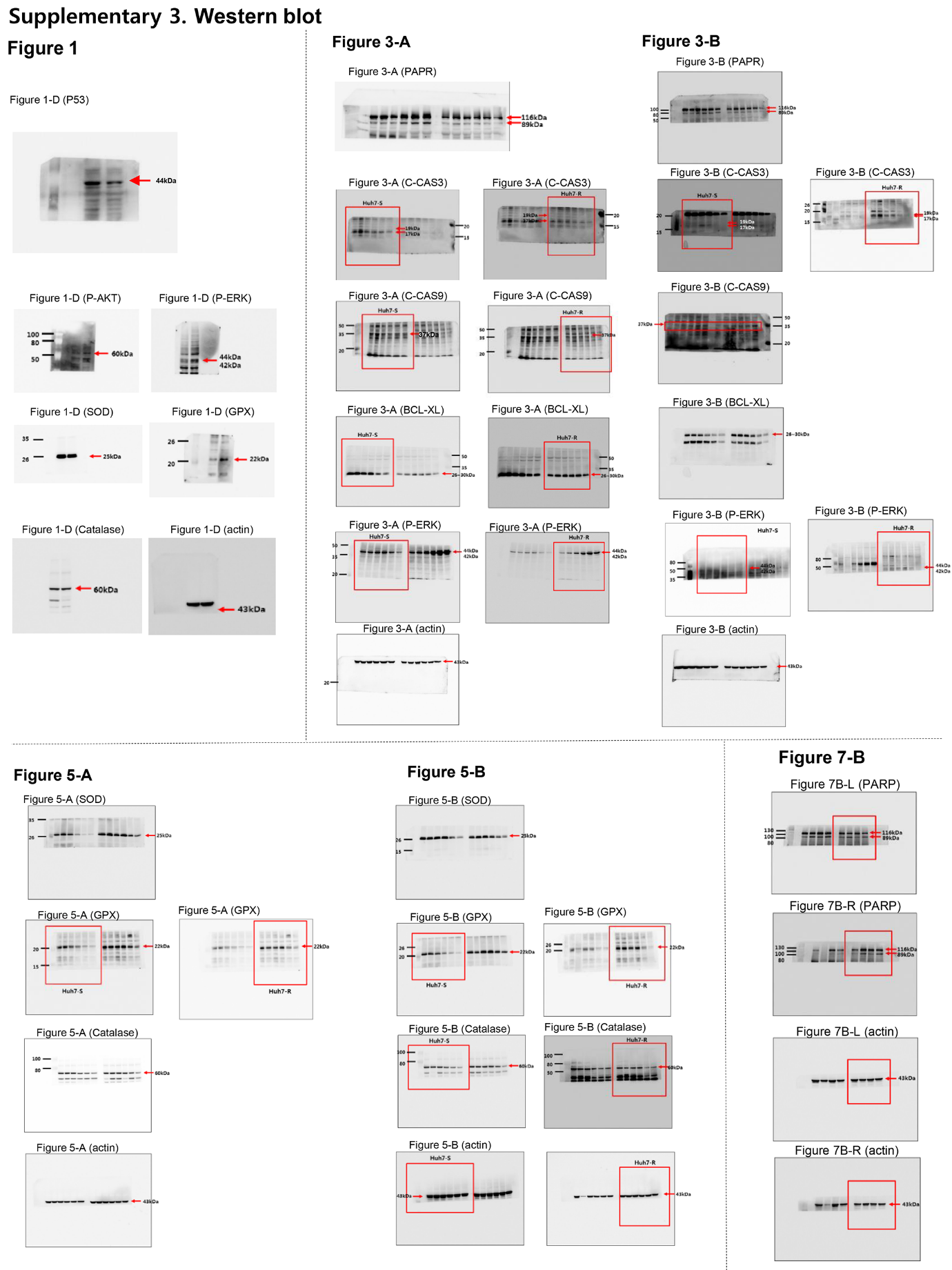

Supplement: Supplementary file 1 — Supplementary Information 1. [file 41598_2020_79536_MOESM1_ESM.docx]
